# Analysis of phosphorylation of human heat shock factor 1 in cells experiencing a stress

**DOI:** 10.1186/1471-2091-6-4

**Published:** 2005-03-11

**Authors:** Toumy Guettouche, Frank Boellmann, William S Lane, Richard Voellmy

**Affiliations:** 1 Biochemistry and Molecular Biology, University of Miami, Miller School of Medicine, 1011 N.W. 15th Street, Miami, FL 33136, USA; 2 Microchemistry & Proteomics Analysis Facility, Harvard University, 16 Divinity Avenue, Cambridge, MA 02138, USA

## Abstract

**Background:**

Heat shock factor (HSF/HSF1) not only is the transcription factor primarily responsible for the transcriptional response of cells to physical and chemical stress but also coregulates other important signaling pathways. The factor mediates the stress-induced expression of heat shock or stress proteins (HSPs). HSF/HSF1 is inactive in unstressed cells and is activated during stress. Activation is accompanied by hyperphosphorylation of the factor. The regulatory importance of this phosphorylation has remained incompletely understood. Several previous studies on human HSF1 were concerned with phosphorylation on Ser^303^, Ser^307 ^and Ser^363^, which phosphorylation appears to be related to factor deactivation subsequent to stress, and one study reported stress-induced phosphorylation of Ser^230 ^contributing to factor activation. However, no previous study attempted to fully describe the phosphorylation status of an HSF/HSF1 in stressed cells and to systematically identify phosphoresidues involved in factor activation. The present study reports such an analysis for human HSF1 in heat-stressed cells.

**Results:**

An alanine scan of all Ser, Thr and Tyr residues of human HSF1 was carried out using a validated transactivation assay, and residues phosphorylated in HSF1 were identified by mass spectrometry and sequencing. HSF1 activated by heat treatment was phosphorylated on Ser^121^, Ser^230^, Ser^292^, Ser^303^, Ser^307^, Ser^314^, Ser^319^, Ser^326^, Ser^344^, Ser^363^, Ser^419^, and Ser^444^. Phosphorylation of Ser^326 ^but none of the other Ser residues was found to contribute significantly to activation of the factor by heat stress. Phosphorylation on Ser^326 ^increased rapidly during heat stress as shown by experiments using a pSer^326 ^phosphopeptide antibody. Heat stress-induced DNA binding and nuclear translocation of a S326A substitution mutant was not impaired in HSF1-negative cells, but the mutant stimulated HSP70 expression several times less well than wild type factor.

**Conclusion:**

Twelve Ser residues but no Thr or Tyr residues were identified that were phosphorylated in heat-activated HSF1. Mutagenesis experiments and functional studies suggested that phosphorylation of HSF1 residue Ser^326 ^plays a critical role in the induction of the factor's transcriptional competence by heat stress. PhosphoSer^326 ^also contributes to activation of HSF1 by chemical stress. To date, no functional role could be ascribed to any of the other newly identified phosphoSer residues.

## Background

Phosphorylation emerged as a major post-translational mechanism that is well suited for effecting a rapid change in the activity of a transcription factor in response to an extracellular signal [1,2]. During periods of physical or chemical stress, transcription of genes encoding cytoprotective heat shock or stress proteins (HSPs) is increased. This enhanced expression is primarily mediated by heat shock factor 1 (HSF1) in vertebrate cells or by a homologous factor (HSF) in non-vertebrate cells. HSF/HSF1 is continuously present in cells but is only activated when the cells experience a stress. It was long known that HSF/HSF1 is hyperphosphorylated in stressed cells [3-5].

Activation of human HSF1 occurs in at least two steps. A first step results in formation of factor homotrimers that are capable of binding so-called heat shock element (HSE) sequences present in *hsp *genes but essentially lack transcriptional activity. In a second step, these HSF1 homotrimers are converted to a transcriptionally competent form [6-8]. In cells exposed to heat, acquisition of HSE DNA-binding activity was observed to precede hyperphosphorylation of HSF1 [9]. This result suggested that hyperphosphorylation could play a regulatory role in the second activation step that renders the factor transactivation-competent. Several additional observations are compatible with the hypothesis that hyperphosphorylation of HSF1 is required for or enhances induction of the transcriptional competence of the factor: (i) To the extent this was examined, all conditions that resulted in activation of HSF1 also induced hyperphosphorylation of the factor. (ii) Conversely, compounds such as salicylate, indomethacin, menadione and hydrogen peroxide that were only capable of triggering the first step of HSF1 activation also failed to prompt factor hyperphosphorylation [8,10,11]. (iii) Inhibitors of Ser/Thr protein kinases reduced, and inhibitors of Ser/Thr phosphatases enhanced, HSF1 activity [11-17]. For the inhibitors investigated it was found that they did not affect HSF1 DNA-binding activity [11] (see also [18]).

To date, stress-induced phosphorylation of HSF/HSF1 has not been comprehensively analyzed. However, phosphorylation of Ser^230 ^of human HSF1 was reported to contribute to heat activation of the factor by enhancing its transcriptional competence [19]. It was also proposed that phosphorylation of Thr^142 ^of human HSF1 may be essential for factor activity [20]. Furthermore, several HSF/HSF1 residues whose phosphorylation repressed factor activity were identified [9,21-30]. In human HSF1 these residues are Ser^303^, Ser^307 ^and Ser^363^. The present study sought to combine systematic mutagenesis and physical analyses to provide a broad accounting of phosphorylation of HSF1 in heat-stressed cells.

## Results

### Validation of a transactivation assay for testing HSF1 mutants

**Figure 1:**
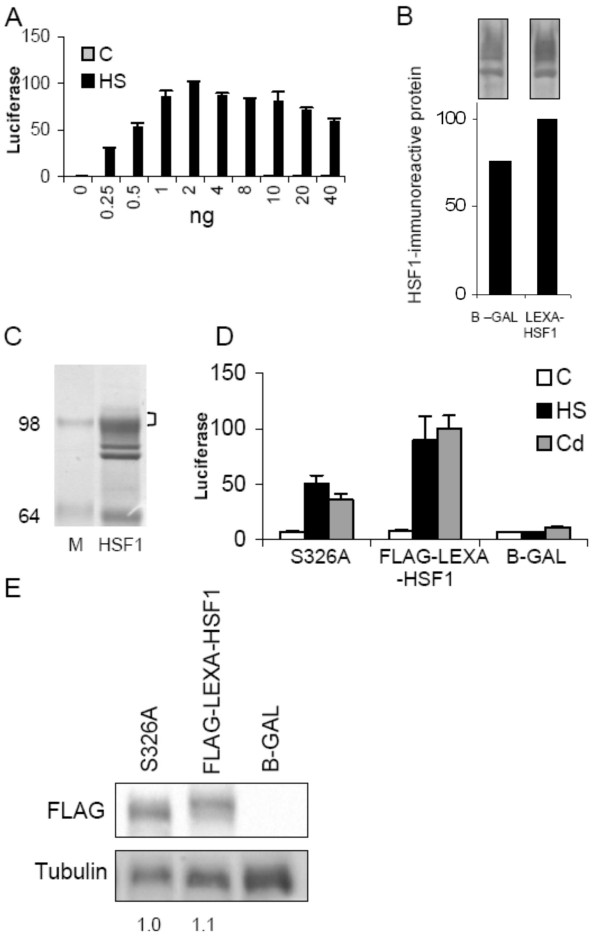
**Validation of the transactivation assay used, transactivation analysis of mutant S326A, and isolation of FLAG-HSF1 for phosphorylation analyses**. (**A**) Transactivation assay of cells in 96-well dishes co-transfected with reporter gene mixture (LEXA-fLUC and pRL-CMV) and 0–40 ng LEXA-HSF1. C: Control unheated cells; HS: Cells subjected to standard heat-treatment for 30 min at 44°C. (**B**) Relative amounts of HSF1-immunoreactive protein in cells transfected with 0.5 ng β-galactosidase expression construct (B-GAL) or 0.5 ng LEXA-HSF1. Extracts prepared one day after transfection were analyzed by anti-HSF1 western blot. A quantitation of the HSF1 signals, shown in the inserts on top, is presented. Note that the insert only shows the relevant (scanned) portion of the blot, i.e., polypeptide sizes from about 60–100 kD. HSF1 forms appear as several closely spaced bands above a single band representing a nonspecific signal. (**C**) Coomassie Blue-stained gel containing immune-isolated FLAG-HSF1. M: markers having molecular weights in kD as indicated on the left. Note that the cluster of bands appearing at and below the 98 kD marker was identified as HSF1 forms by a parallel anti-HSF1 western blot (not shown). (**D**) Transactivation assay comparing activities of HSF1 forms in cells co-transfected with luciferase reporter gene mixture and expression constructs (0.5 ng) for FLAG-LEXA-HSF1 substitution mutant S326A, parent FLAG-LEXA-HSF1 or B-GAL. One day after transfection, cells were exposed either to 300 μM CdCl_2 _for 2 h (Cd) or to standard heat treatment (HS), or were left untreated (C). Relative firefly luciferase activities assayed 6 h after treatments are shown. (**E**) FLAG western blot of parallel (heat-treated) cultures from the experiment analyzed under C that compares expression levels of FLAG-LEXA-HSF1 mutant S326A and parent FLAG-LEXA-HSF1 one day after transfection. The tubulin signal was used as loading control. The numbers below the blots represent a quantitative comparison of the mutant S326A and parent FLAG-LEXA-HSF1 signals in the FLAG blot.

In chimeric transcription factor LEXA-(human)HSF1 the DNA-binding domain (residues 1–78) of the 529-residue human HSF1 polypeptide is substituted with that of bacterial repressor LEXA (residues 1–87) [7]. LEXA-HSF1 is known from previous studies to be regulated similarly as HSF1 [7]. Transactivation by LEXA-HSF1 was assessed by dual luciferase assay of cells co-transfected with a firefly luciferase gene responsive to LEXA-HSF1 (LEXA-fLUC) and a constitutively expressed *Renilla *luciferase gene (pRL-TK or pRL-CMV). Reporter activity was expressed as the ratio of firefly and *Renilla *luciferase activities. To permit the identification of mutants of LEXA-HSF1 with only minor functional impairments, the transactivation assay needed to sensitively detect changes in transcription factor activity. To find out whether the assay had this capability under the chosen experimental conditions, 96-well cultures were transfected with different amounts of expression construct LEXA-HSF1 (Figure 1A). At amounts below about 2 ng/culture, reporter activity increased proportionally with the amount of expression construct transfected. Hence, at these low DNA concentrations changes in LEXA-HSF1 activity were certain to be reflected in proportional changes in reporter activity. Most subsequent transfections were carried out with 0.5 ng or less of expression construct per 96-well culture. Transfection with 0.5 ng of construct LEXA-HSF1 resulted in a 30% increase in total HSF1 concentration as estimated by western blot one day after transfection (Figure 1B). Assuming a typical transfection efficiency of 20–40%, this result implied that transfected cells on average expressed comparable amounts of LEXA-HSF1 and endogenous HSF1. Hence, under these conditions, under which an exogenous HSF1 form was not or was only minimally overexpressed, the regulatory environment encountered by the exogenous HSF1 form was likely to be similar to that to which endogenous factor is exposed. This is a departure from several earlier studies relating to HSF1 phosphorylation, including one from our own group [28], in which studies exogenous HSF1 forms were substantially overexpressed. A known result of such overexpression is that a significant fraction of exogenous factor accumulates as DNA-binding trimers in the absence of a stress [7]. At the low levels of expression construct transfected in the assays used herein, this result did not occur (as documented, e.g., by the experiment shown in Figure 3A).

### Alanine scan of LEXA-HSF1

Substitution mutants of LEXA-HSF1 or FLAG-LEXA-HSF1 (FLAG-tagged LEXA-HSF1) were prepared that collectively covered all 92 Ser, Thr and Tyr residues of the HSF1 sequence present in LEXA-HSF1. Transactivation assays were carried out to compare transcription-enhancing abilities of mutant and parent factors in cells that either had been exposed to a 44°C/30 min heat treatment or had not been heat-treated. It is noted that this heat treatment resulted in only minimal cell death. Results revealed that most mutants retained the strong heat inducibility of the respective parent factor (Table 1). Nine mutants (highlighted) had an induced activity that was lower than that of parent factor by more than two average standard deviations (2 × 10.3%). Five of these mutants were only modestly impaired, but four mutants, S326A/T328A, T400A/S(403/404)A, S(457/458/461)A and T511A/S513A, had substantially reduced activities. Mutants S326A/T328A and T511A/S513A were expressed to normal levels. However, mutant T400A/S(403/404)A accumulated to a noticeably lower level, and mutant S(457/458/461)A was cleaved proteolytically (western blot data not shown). These results suggested the possibility that phosphorylation of residues 326, 328, 511 and/or 513 played an important role in activation of HSF1 by heat stress. Mutants S(303/307)A and S307A were about 150% and 50%, respectively, more active than parent factor in heat-treated cells (Table 1).

**Table 1:** Alanine scan of LEXA-HSF1

Construct	Relative luciferase			
	
	C	SD	HS	SD
LEXA-HSF1	0.50	0.1	100	13.5
LEXA-HSF1(F)	0.32	0.1	100	6.3
T97A	0.83	0.3	122	5.0
T120A/S121A(F)	0.59	0.1	115	4.7
S123A/T124A	0.65	0.6	95	6.6
S127A	0.50	0.0	84	5.1
S136A/T138A(F)	0.23	0.1	112	11.6
T142A(F)	0.22	0.0	83	11.0
S156A(F)	0.29	0.0	102	6.1
S(174/195/199)A	0.30	0.0	105	8.2
**S216A**	**0.52**	**0.1**	**77**	**10.5**
S218A(F)	0.31	0.0	117	9.5
**S221A(F)**	**0.64**	**0.4**	**70**	**8.3**
Y225A/S(226/230)A(F)	0.39	0.0	92	5.8
S(237/241/244)A/Y240A	0.54	0.1	126	6.4
Y247A/S(248/249)A(F)	0.72	0.1	127	2.2
S(250/251)A/Y253(F)	1.21	0.3	99	16.6
S(260/261)A	0.84	0.2	119	19.1
S266A/T269A(F)	0.95	0.3	83	11.8
**S275A**	**1.05**	**0.3**	**75**	**5.5**
S279A	0.51	0.0	103	24.7
S290A	0.32	0.0	101	13.5
S291A	1.43	0.4	80	4.1
S292A	1.47	0.6	84	3.1
S303A	0.97	0.3	81	13.9
S307A(F)	0.36	0.0	148	33.7
S(303/307)A(F)	1.27	0.5	249	16.9
S314A(F)	0.34	0.0	131	18.3
S(319/320)A/T323A	0.83	0.2	78	10.8
**S326A/T328A**	**2.15**	**1.0**	**46**	**1.9**
S333A(F)	0.37	0.0	103	9.3
S338A(F)	0.51	0.1	88	10.1
S344A(F)	0.47	0.1	101	13.4
T346A(F)	0.60	0.1	108	15.0
T349A(F)	0.35	0.1	95	12.6
T(355/357)A(F)	0.34	0.0	129	12.6
S(363/368)A/T(367/369)A(F)	1.05	0.6	92	14.4
S375A	2.13	1.9	73	13.6
S385A(F)	0.57	0.1	121	5.7
S393A	1.38	0.6	83	14.9
**T400A/S(403/404)A(F)**	**0.57**	**0.2**	**63**	**2.9**
T411A/S412A(F)	0.57	0.0	85	6.3
S(419/421)A/T423A(F)	0.48	0.0	101	6.7
**S428A**	**0.88**	**0.1**	**75**	**9.6**
S(434/435/438)A	1.39	0.1	81	12.9
S444A	1.27	0.2	88	13.2
**S(457/458/461)A(F)**	**0.49**	**0.1**	**7**	**0.3**
Y468A/T469A	2.12	1.9	103	16.6
S(480/485)A/T483A	0.47	0.1	80	4.0
S(498/501)A/Y499A	1.08	0.1	80	9.2
**T511A/S513A(F)**	**0.44**	**0.1**	**22**	**1.0**
T516A/S518A(F)	0.87	0.1	78	8.1
T527A/S529A	1.14	0.2	91	4.6
S121A	1.73	0.2	106	12.1
S230A	1.64	0.3	101	22.7
S319A	0.60	0.1	105	14.3
S320A	0.36	0.1	91	12.8
T323A	1.97	0.5	102	17.6
S326A	1.66	0.3	40	8.1
S419A	1.67	0.2	104	17.8

C: not-heated; SD: standard deviation; HS: heat-treated. Transactivation assays were carried out as discussed in Methods and Results. (F) refers to the presence of an amino-terminal FLAG tag (see Methods). The activities of FLAG-tagged mutants are expressed relative to the activity of FLAG-LEXA-HSF1, and those of not-FLAG-tagged mutants to the activity of LEXA-HSF1.

### Residues phosphorylated in HSF1 isolated from heat-treated cells

In a first series of experiments, cultures transfected on the previous day with expression construct FLAG-HSF1 were pre-equilibrated with ^32^PO_4 _and then heat-treated for 45 min at 44°C. Exogenous HSF1 was immunoprecipitated using an anti-FLAG resin. In a stained, high-resolution Tris-Tricine SDS-PAGE gel (Figure 1C), immune-isolated HSF1 appeared as two sharp bands and a slower migrating diffuse region that contained the most highly phosphorylated forms (that were also most intensely radiolabeled; data not shown). Protein from the latter region (see bracket in Figure 1C) was subjected to trypsin or trypsin/chymotrypsin digestion. Phosphopeptides were separated by HPLC, tentatively identified by mass spectrometry (MALDI-MS) and confirmed by regular and/or radiochemical sequencing. In subsequent experiments, peptides from unlabeled, purified FLAG-tagged HSF1 digested with trypsin, trypsin/chymotrypsin, or endoproteinases Glu-C or AspN were analyzed by tandem mass spectrometry (LC/MS/MS). These analyses resulted in an unambiguous identification of phosphoserines at positions 121, 230*, 292, 303*, 307*, 314, 319, 326, 344, 363*, 419, and 444 of the HSF1 sequence (Table 2; *previously reported sites). No phosphorylated Thr or Tyr residues were discovered. This analysis covered >90% of the HSF1 sequence. No information was obtained about residues 176–184, 202–206, 225–227, 241–256, 270–284 and 427–432.

**Table 2:** Residues phosphorylated in HSF1 from heat-treated cells.

Peptide	N	Phospho-residue	Dig.
VEEA**pS**PGRP**pS**SVDTLL**pS**PTALIDSILR	4	314, 319, 326	Trp.
VKEEPP**pS**PPQ**pS**PR	2	303, 307	Trp.
GHTDTEGRPP**pS**PPPTSTPEK	3	363	Trp.
VVHIEQGGLVKPERDDTEFQHPCFLR	1	(97)	Trp.
GHTDTEGRPP**pS**PPPTSTPEK*	2	363	Trp.
VKEEPP**pS**PPQ**pS**PR*	2	303, 307	Trp.
QF**pS**LEHVHGSGPY*	1	230	Trp.
VEEA**pS**PGRP**pS**SVDTLL**pS**PTALIDSILR*	3	314, 319, 326	Trp, Chytrp.
VEEASPGRPSSVDTLL**pS**PTALIDSILR	2	326	Trp, Chytrp.
VEEA**pS**PGRPSSVDTLL**pS**PTALIDSILR	2	314, 326	Trp, Chytrp.
VKEEPP**pS**PPQSPR	1	303	Trp, Chytrp.
GHTDTEGRPP**pS**PPPTSTPEK	2	363	Trp, Chytrp.
KVT**pS**VSTLKS	1	121	Trp, Chytrp.
PLSS**pS**PLVR	1	292	Trp, Chytrp.
**pS**LEHVHGSGPY	1	230	Trp, Chytrp.
DARGHTDTEGRPP**pS**PPPTSTPEKCLSVACL	2	363	AspN
DERPLSS**pS**PLVRVK	2	292	AspN
DLF**pS**PSVTVP	1	419	AspN
DSSLASIQELL**pS**PQEPPRPPEAENSSP	1	444	AspN
DTLL**pS**PTALI	1	326	AspN
PASVTALTDARGHTDTEGRPP**pS**PPPTSTPE	1	363	Glu-C
LL**pS**PQEPPRPPEAEN	1	444	Glu-C
SEPAPA**pS**VTALTDARGHTDTE	1	344	Glu-C
A**pS**PGRPSSVDTLL**pS**PTALID	2	314, 326	Glu-C
LF**pS**PSVTVPD	1	419	Glu-C

N: Unambiguously identified phosphoresidues are bolded and are preceded by p. Where identification was ambiguous, candidate residues are underlined. Dig.: proteinase used for digestion of HSF1. *Radiolabeled peptide. Trp: trypsin; Chytrp: chymotrypsin. For experimental details relating to analysis of HSF1 phosphopeptides and identification of phosphorylated residues see Methods.

### Phosphorylation of HSF1 residue Ser^326^

Based on results from the above-described alanine scan HSF1 residues 326, 328, 511 and/or 513 were considered potential sites for regulatory phosphorylation. Of these residues only Ser^326 ^was actually found phosphorylated in HSF1 from heat-shocked cells. Not all residues that were identified as targets of phosphorylation by mass spectrometry and/or sequencing had been substituted individually in the earlier alanine scan. To rule out the possibility that an effect of substitution of a phosphorylated residue had somehow been masked or otherwise modulated (in the case of Ser^326^) by other substitutions present in the same mutant, single substitutions were prepared and examined in the transactivation assay. All substitutions of phosphorylated serines except for the S326A substitution displayed heat-induced activities comparable to that of parent factor (Table 1, bottom). The S326A substitution was only about 40% as active as the parent factor (35–55% in individual experiments). To test whether substitution of Ser^326 ^affected not only heat-induced but also chemically induced HSF1 activity, the activity of the S326A mutant was tested in cells exposed to CdCl_2_. Induction by CdCl_2 _was found to be similarly impaired as induction by heat (Figure 1D). Note that this reduced activity phenotype was not due to a reduced level of accumulation of the mutant as evidenced by the anti-FLAG western blot shown in Figure 1E. Nucleotide sequencing of the entire S326A-coding sequence confirmed that it did not contain any additional mutation. Furthermore, a second copy of mutant S326A obtained in a separate mutagenesis experiment had a similarly impaired stress-induced activity as the original copy.

### Rapid phosphorylation of HSF1 residue Ser^326 ^during heat stress

**Figure 2:**
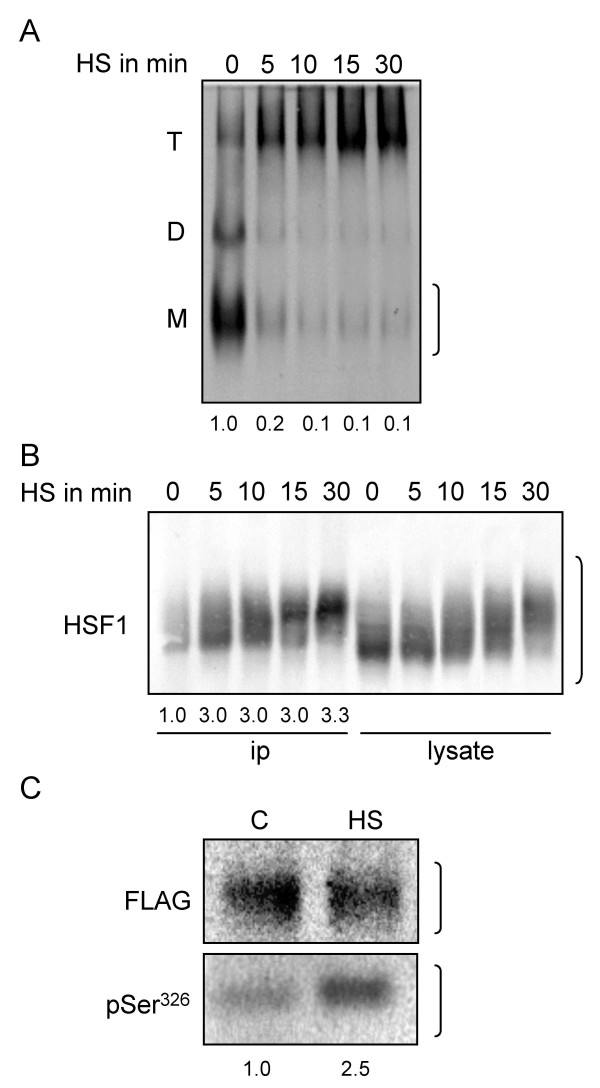
**Heat-induced oligomerization and phosphorylation of HSF1**. (**A**) Native anti-HSF1 blot showing heat-induced oligomerization of endogenous HSF1 in response to heat treatment at 44°C (HS) for the times indicated on top of the blot. T: HSF1 trimers; D: heterodimers; M: monomers. (**B**) Parallel cultures to those used in A for an analysis of HSF1 oligomerization were employed here for an examination of global or Ser^326^-specific phosphorylation of endogenous HSF1. The anti-HSF1 western blot shown reports the distribution of HSF1 forms of different apparent size (reflecting different levels of phosphorylation) in extract samples (lysate) or in protein immunoprecipitated from the same extracts by pSer^326 ^antibody (ip). The portion of the blot depicted only shows protein signals larger than about 70 kD. (**C**) Detection of heat-induced phosphorylation of Ser^326 ^by western blot using anti-pSer^326 ^antibody. Parallel cultures were transfected with small amounts of FLAG-HSF1. One day later, the cultures were either left untreated (C) or were heat-treated for 30 min at 44°C (HS) and were processed for western blot immediately following the heat treatment. The anti-pSer^326 ^blot reports on induction of Ser^326 ^phosphorylation by heat, and the parallel anti-FLAG blot shows that similar amounts of FLAG-HSF1 were compared in the anti-pSer^326 ^blot. Data from densitometry are shown below the blots. Brackets shown on the side of blots indicate lengths of regions scanned. In A, the monomer signal was quantitated.

A phosphopeptide antibody (referred to below as pSer^326 ^antibody) was prepared to specifically monitor phosphorylation of Ser^326^. Heat-induced rates of overall phosphorylation of HSF1 (i.e., phosphorylation of all available sites), of specific phosphorylation of Ser^326 ^and of factor oligomerization were compared in the experiment shown in Figure 2 (panels A and B). Cultures were either not heat-treated or heat-treated at 44°C for 5, 10, 15 or 30 min. HSF1 trimerization was assessed by native anti-HSF1 blot. As best seen by following the disappearance of the monomeric form, most HSF1 was or was in the process of becoming oligomeric within 5 min of heat treatment (Figure 2A). For examining overall and Ser^326^-specific phosphorylation, HSF1 was immunoprecipitated using pSer^326 ^antibody. Immunoprecipitated HSF1 (Figure 2B, "ip" lanes) and HSF1 in extract ("lysate" lanes) were detected by anti-HSF1 western blot. To better resolve different HSF1 species, electrophoresis was continued until the 50 kDa marker had migrated to the end of the gel (only the region containing HSF1 forms, i.e. from about 70 kD protein size, is shown in Figure 2B). Results suggested that unstressed cells contained a small amount of HSF1 that could be immunoprecipitated by the pSer^326 ^antibody ("ip", first lane). Note that, as would have been expected, the most rapidly migrating immunoprecipitated HSF1 form was slower than the fastest species present in extract (compare 0-min "ip" and "lysate" lanes). Upon heat treatment of the cells, amounts of HSF1 precipitatable from extracts by the pSer^326 ^antibody increased about 3 fold (see quantitation below "ip" lanes). This increase was essentially complete after only 5 min of heat treatment. Overall phosphorylation as revealed by a shift from faster- to slower-migrating HSF1 species occurred more slowly and continued over the entire 30-min course of heat treatment ("lysate" lanes). A comparable gradual shift to slower-migrating species was also observed for pSer^326^-containing HSF1 species ("ip" lanes). Thus, heat treatment appeared to result in a substantial increase in the level of phosphorylation of Ser^326^. This increase in phosphorylation occurred about as rapidly as factor oligomerization and preceded phosphorylation of most other sites.

**Figure 3:**
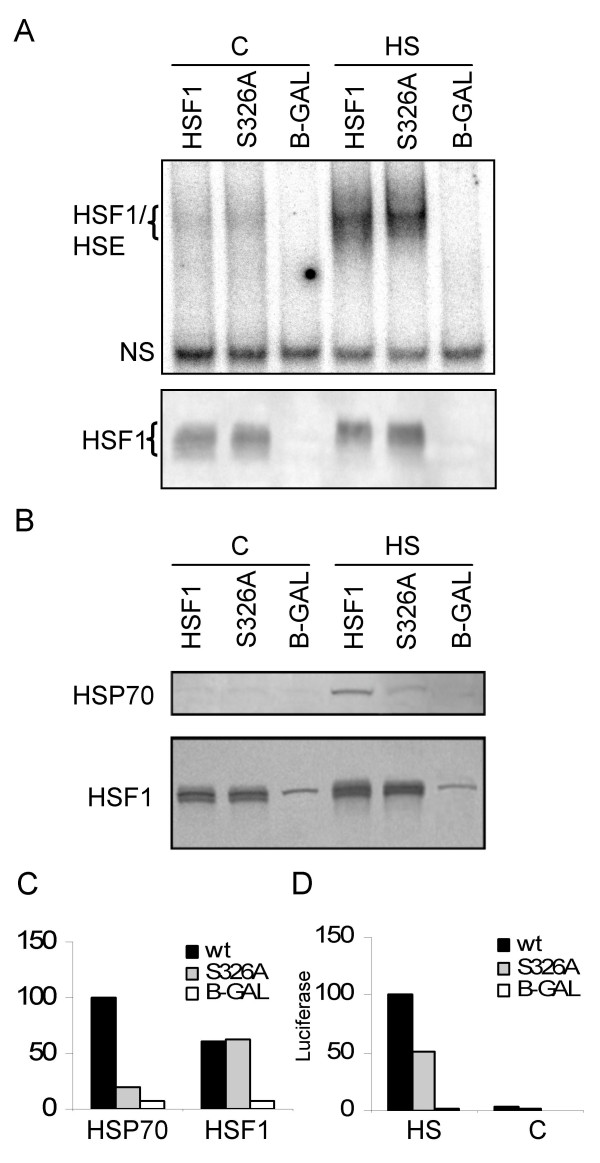
**Comparative analyses in HSF1-negative mouse embryo fibroblasts**. (**A**) Comparison of DNA-binding abilities of HSF1 and HSF1 mutant S326A. An electrophoretic mobility shift assay was carried out using an HSE DNA probe and extracts from heat-treated or not-heat-treated cells that had been transfected one day earlier with the constructs indicated on top of the gel. An anti-HSF1 western blot of the same samples reporting on the relative levels of expression of HSF1 forms from the different constructs is shown below the gel. HSF1/HSE: HSF1-DNA complex; NS: nonspecific signal, serving as a loading control for the group of samples from not-heated cells, and, independently, for the group of samples from heat-treated cells; HS: heat-treated for 30 min at 43°C; C: not heat-treated. (**B**) Heat-induced transactivation of endogenous *hsp70 *gene(s) in cells transfected with small amounts of the constructs indicated above the blots and with luciferase reporters HSP70-fLUC and pRL-CMV. One day after transfection, cultures were either left untreated (C) or were heat-treated for 30 min at 43°C (HS). Extracts were prepared after 6 h of further incubation at 37°C and were used for western blots. The top blot was probed with an antibody recognizing HSP70, whereas the bottom blot was probed with HSF1 antibody. The bottom blot demonstrates that mutant and wildtype HSF1 forms accumulated to similar levels. Note the presence of a weak nonspecific signal present in all lanes that happened to co-migrate with the transfected HSF1 forms. (**C**) Quantitative comparisons of data of B. (**D**) Relative luciferase reporter activities in the same extracts that were analyzed for HSP70 in B. Representative results from one of several independent experiments are shown in this Figure.

Heat-induced phosphorylation of Ser^326 ^was confirmed by a second experiment, in which HSF1 phosphorylated at Ser^326 ^was detected by anti-pSer^326 ^western blot. Because of the low avidity of the antibody, HSF1 needed to be enriched prior to western blot. Large cultures (in 100 mm plates) were transfected with small amounts of expression construct FLAG-HSF1. One day later, half of the cultures were heat-treated at 44°C for 30 min, and tagged HSF1 was immunoprecipitated using an anti-FLAG resin. Immune-isolated material was then analyzed by western blot using pSer^326 ^and FLAG antibodies (Figure 2C). Recovery of FLAG-HSF1 from heat-treated and untreated cells was comparable (anti-FLAG blot on top). A substantially larger fraction (2.5 fold) of factor from heat-treated cells than from not-heat-treated cells reacted with the pSer^326 ^antibody.

### Phosphorylation of Ser^326 ^specifically enhances HSF1 transactivation competence

To examine the effects of substitution of Ser^326 ^in an otherwise wildtype HSF1 background, use was made of an HSF1-negative mouse cell line prepared previously by McMillan et al. [31]. Analyses were carried out one day after transfection of expression constructs for HSF1, substitution mutant S326A (in wildtype HSF1 not LEXA-HSF1 background) and control protein β-galactosidase. First, it was examined whether phosphorylation of Ser^326 ^affected the first step of HSF1 activation, which step involves acquisition of HSE DNA-binding activity and nuclear localization. Electrophoretic mobility shift assay revealed that HSE DNA-binding activities of wildtype HSF1 and mutant S326A were heat-induced to similar levels (Figure 3A). Nuclear localization was assayed by standard fractionation of cell extracts and anti-HSF1 western blot. Comparable amounts of wildtype HSF1 and mutant S326A were present in the nuclear fraction of heat-treated cells (data not shown). Thus, phosphorylation of Ser^326 ^did not affect the first step of HSF1 activation. To probe the second activation step, i.e., acquisition of transactivation competence, HSF1-negative cells were co-transfected with the above expression constructs and with reporter constructs HSP70-fLUC and rLUC. Cultures either were left untreated or were heat-treated at 43°C for 30 min and further incubated for 6 hours. Transactivation competence was estimated by western blot of endogenous HSP70 (Figure 3, panels B and C) and by luciferase assay (Figure 3D). Heat-induced expression of endogenous HSP70 was reduced by 80% in the cells expressing the S326A mutant of HSF1 when compared to the cells expressing wildtype HSF1. The transfected luciferase reporter was reduced by 50%. The greater effect on HSP70 expression is likely explained by the difference in RNA/protein stability between HSP70 and luciferase.

## Discussion

The present study attempted for the first time to examine phosphorylation of HSF1 in cells exposed to a stress in a comprehensive fashion. Although HSF/HSF1 is activated in cells exposed to various types of stressful events, in the interest of being able to complete a thorough analysis, we decided to focus on phosphorylation of HSF1 in cells responding to a single type of stress, i.e., a heat stress. Our study identified twelve serine residues in human HSF1 that are phosphorylated in heat-stressed cells. Eight of these residues represent phosphorylation sites that were not previously known, i.e., Ser^121^, Ser^292^, Ser^314^, Ser^319^, Ser^326^, Ser^344^, Ser^419^, and Ser^444^. Phosphorylation of all residues previously found to be phosphorylated *in vivo*, i.e., Ser^230^, Ser^303^, Ser^307 ^and Ser^363^, was confirmed. No phosphorylation of Thr^142 ^or any other Thr or Tyr residue was observed.

Because the importance of the various phosphorylation events for the activation of human HSF1 could not be predicted, an effort was made to ensure that the basic transactivation assay used in the present study was capable of reporting even relatively minor impairments in the activity of HSF1 mutants as well as examined the exogenous HSF1 forms under conditions that differed as little as possible from those encountered by endogenous factor. Although HSF1-deficient mouse cells were available and were used in later experiments, our initial goal was to identify mutants of human HSF1 that were functionally deficient in human cells. Therefore, mutants were prepared in the LEXA-HSF1 background, allowing us to test effects of mutations in cells containing endogenous HSF1. Because hyperphosphorylation was expected to affect HSF1 transcriptional competence rather than HSE DNA-binding ability, use of an HSF1 form identical to HSF1 except for a substituted DNA-binding domain appeared justified. For obvious reasons, an HSF1 mutant with an impaired activity was not available when assay conditions needed to be established. In the absence of such a mutant, assay conditions were defined, under which reporter activity increased proportionally with amounts of LEXA-HSF1 expression construct transfected. Under the chosen conditions, exogenous HSF1 (i.e., LEXA-HSF1) was expressed at a comparable level as endogenous HSF1. Hence, these conditions also satisfied our second criterion that was to assay exogenous HSF1 in an intracellular situation that closely resembled that encountered by endogenous HSF1. The importance of the latter criterion is exemplified by the previous observation that substantially overexpressed exogenous HSF1 is trimeric and DNA binding in the absence of a stress, whereas endogenous HSF1 is not trimeric and DNA binding under the same conditions [7]. In the transactivation assays used in the present study, exogenous HSF1 did not specifically bind DNA in the absence of a stress.

The above-discussed differences between the transactivation assay used in the present study and assays employed in earlier studies provide a ready explanation for the observed differences in phenotypes of mutants S(303/307)A and S307A. The latter substitutions (also in LEXA-HSF1 background) had been examined in a previous study by our laboratory and were found to be active in the absence of a stress [28] (see also [9,23,25]). In this earlier study, HSF1 forms were substantially overexpressed, resulting in accumulation of homotrimeric factors in the absence of a stress. Therefore, the experiments were only capable of assessing effects of mutations on HSF1 transcriptional competence. When examined using the assay of the present study, in which assay oligomerization of exogenous HSF1 forms is regulated, the Ser^307 ^and Ser^303^/Ser^307 ^substitutions could be expected to be inactive in the absence of a stress, provided that the mutations only affected HSF1 transactivation competence and not also oligomerization. As shown in Table 1, this expected result was observed. In heat-stressed cells, however, the Ser^307 ^and Ser^303^/Ser^307 ^substitutions exceeded the activity of the parent factor. This finding is consistent with a role of phosphorylation at Ser^303 ^and Ser^307 ^in down-modulation of HSF1 activity during a heat stress or, more likely, during recovery from the stress. Such a role has been proposed previously by others (e.g., [23,27,32]). Also compatible with this hypothesis is that, in a tryptic digest of HSF1 isolated from cells pulse-labeled with ^32^PO_4 _during a 44°C/45 min heat treatment, peptide 297–309 was among the most intensely radiolabeled peptides (not shown). This finding implied that phosphorylation of Ser^303 ^and/or Ser^307 ^occurred during heat treatment. Hietakangas et al. recently confirmed that Ser^303 ^is inducibly phosphorylated by western blot experiments using a phosphopeptide antibody recognizing pSer^303 ^[33]. As Ser^303 ^phosphorylation may require prior phosphorylation of Ser^307 ^[23], phosphorylation of Ser^307 ^is likely also heat-inducible.

The present study identified HSF1 residue Ser^326 ^as a dominant target of regulatory phosphorylation during activation of the factor by a heat stress. Luciferase reporter assays indicated that phosphorylation of Ser^326 ^causes the heat-induced activity of HSF1 to at least double. The transactivation assays in which endogenous HSP70 was used as the endpoint revealed that this enhancement of HSF1 activity translates into a fivefold increase in accumulation of HSP70. While our study did not address this issue, it seems likely that the observed five-fold enhancement of HSP70 expression resulting from phosphorylation of Ser^326 ^is physiologically important. A previous study demonstrated that a five to eight fold impairment in heat-induced HSP70 expression led to substantially diminished thermotolerance of the affected mouse embryo fibroblast cells [34]. Phosphorylation of Ser^326 ^also appears to significantly contribute to HSF1 activity induced in cells stressed by exposure to CdCl_2_.

Our analyses suggested that, individually, phosphorylation of none of the other residues identified as being phosphorylated in heat-treated cells significantly contributes to HSF1 activity. The possibility was considered that phosphorylation of several of these residues may be required for producing a clearly detectable effect. Although this possibility was not examined exhaustively, several combinations of substitutions including or excluding the S326A substitution were tested by transactivation assay (data not shown). These experiments failed to uncover evidence for a functional effect of phosphorylation on residues other than Ser^326^. It is puzzling that HSF1 is phosphorylated on a number of residues (e.g., Ser^121^, Ser^230^, Ser^292^, Ser^314^, Ser^319^, Ser^344^, Ser^419^, and Ser^444^) whose phosphorylation does not appear to affect factor activity. The possibility cannot be formally ruled out that phosphorylation of some of these residues may reflect artifacts due to differences in phosphorylation of exogenous and endogenous HSF1. A more reasonable explanation may be that this phosphorylation may be relevant under conditions not tested in the present study. Such conditions may include different types of stresses or different levels of stresses used to activate HSF1. They may even relate to differences in the transactivation assays used that may result in preferential assessment of different facets of HSF1 activation. This latter explanation may apply to phosphorylation of Ser^230 ^that was previously reported to contribute to activation of HSF1 by heat stress [19]. Another likely possibility is suggested by the fact that HSF1 not only transactivates HSP genes but also participates in the regulation of several important signaling pathways (e.g., [35-38]). Phosphorylation of Ser residues that appears gratuitous with respect to regulation of HSP expression may affect interactions of HSF1 with components of these other pathways and alter their activity.

In agreement with earlier work, heat stress induced rapid trimerization of HSF1. The substantial enhancement of phosphorylation of Ser^326 ^that was induced by heat stress occurred within a similar time frame. This rapid rate of phosphorylation of Ser^326 ^was commensurate with what was expected for a phosphorylation event that was critical for heat stress activation of HSF1. Most other phosphorylation events that could be monitored by their effect on gel mobility occurred more slowly. Hence, it was possible that some of these later events required prior phosphorylation of Ser^326^. However, that at least some of this phosphorylation occurred independent of Ser^326 ^phosphorylation was suggested by the observation of a heat-induced SDS-PAGE mobility shift for mutant S326A (data not shown).

The present study provides evidence that phosphorylation of Ser^326 ^stimulates the transcription-enhancing activity of HSF1 but not its DNA-binding activity. How this phosphorylation results in increased transcriptional competence of HSF1 remains to be elucidated. The observation that substitution of Ser^326 ^with neither Asp nor Glu reproduced the effect of phosphorylation on factor activity (data not shown) suggested that the mechanism is not based on simple charge repulsion. Perhaps, phosphorylation of Ser^326 ^induces a local conformational change that affects binding of a chaperone complex or another regulatory protein to the nearby regulatory domain that is known to be involved in repression of transcriptional competence [6]. Alternatively, pSer^326 ^may be a critical aspect of a binding site for an unknown co-activator. Identification of the protein kinase that phosphorylates Ser^326 ^in heat-stressed cells would be helpful for determining whether the level of phosphorylation of the residue is actively regulated and, if this were the case, by what stress-induced mechanism. Unfortunately, a search of the sequence within which Ser^326 ^is embedded for protein kinase sites using the NetPhosp program [39] did not provide any useful information about candidate protein kinases.

## Conclusion

The present article is concerned with regulation of human HSF1, which is a key factor mediating the transcriptional response of human cells to physical and chemical stresses and a coregulator of other important signaling pathways (e.g.. [35-38]). HSF1 has even been discovered to regulate aging and age-related disease [40,41]. To arrive at a better description of the mechanisms that enable cells to respond to various stresses by transiently upregulating HSP gene expression, it will be important to learn about the extent to which phosphorylation of HSF1 modulates these responses as well as to discover how phosphorylation/dephosphorylation of HSF1 itself is regulated by stresses. Furthermore, it can be expected that a thorough understanding of regulatory phosphorylation of HSF1 will advance our knowledge about what controls the interactions of this factor with other pathways as well as likely will, through the eventual identification of regulated protein kinases and phosphatases involved in HSF1 phosphorylation and dephosphorylation, lead to the identification of new connections with additional regulatory systems. The present study represents an initial contribution towards these larger goals. Our systematic analysis of HSF1 phosphorylation in heat-stressed cells identified twelve phosphorylated Ser residues, of which eight were not previously known. Mutagenesis and functional experiments revealed that newly identified phosphoSer^326 ^plays an important role in heat activation of HSF1 transcriptional activity as evidenced by the fact that substitution of this residue reduced HSP70 accumulation several fold. Phenotypes for substitutions of Ser^303 ^and Ser^307 ^were observed that are consistent with the previously proposed function of phosphorylation of these residues in HSF1 deactivation. Although no evidence for functional roles of other phosphoserines could be obtained in this study, knowledge of the identity of most or all residues phosphorylated in heat-activated HSF1 should greatly facilitate further directed experiments to test the potential importance of their phosphorylation in the various processes and interactions in which HSF1 is known to participate. It cannot be excluded that through the use of different transactivation assays functions of the latter phosphoresidues in heat regulation of HSF1 activity may be discovered that escaped detection in this study.

## Methods

### Antibodies

PhosphoSer^326^-specific rabbit polyclonal antibody was raised against peptide CSVDTLLpSTAL. The antiserum was positively and negatively affinity-purified on immobilized phosphorylated and unphosphorylated peptide. For immunoprecipitations, purified antibody was cross-linked to a resin using the Seize Primary Immunoprecipitation Kit (Pierce). HSF1 antiserum was from StressGen Biotechnologies; FLAG antibody M5, FLAG resin M2 and tubulin antibody were from Sigma; HSP70 antibody 4G4 was from Affinity Bioreagents. Mouse monoclonal antibody 4G4, which antibody was raised against human Hsp70, also recognizes mouse Hsp70. Antibody signals were detected by chemifluorescence and were quantitated on a Molecular Dynamics Storm system.

### Cell culture

Hela-CAT cells [42] were maintained at 37°C and 5% CO_2 _in DMEM containing 10% fetal bovine serum, 100U/ml penicillin and 100 μg/ml streptomycin. HSF1-negative mouse embryo fibroblasts were grown in supplemented DMEM as described by McMillan et al. [31].

### Expression constructs and site-directed mutagenesis

Sequences coding for complete (human) HSF1 (residues 1–529), LEXA-HSF1 (containing residues 1–87 of LEXA and residues 79–529 of human HSF1) and amino-terminally FLAG-tagged derivatives were subcloned into pcDNA3.1(+) (Invitrogen), placing the sequences under the control of a CMV promoter [7]. These constructs were named HSF1, LEXA-HSF1, FLAG-HSF1 and FLAG-LEXA-HSF1, respectively. For the alanine scan, LEXA-HSF1 and FLAG-LEXA-HSF1, were used as templates for QuikChange^R ^site-directed mutagenesis (Stratagene Instruction Manual). Complementary primer pairs replaced single or multiple Ser, Thr or Tyr codons with Ala codons. Potential mutant genes were characterized by restriction analysis, expression in a rabbit reticulocyte lysate-based transcription and translation system (T7 Quick TNT system, Promega) and nucleotide sequence analysis. Several mutations were also introduced into construct HSF1 using the same approach. The β-galactosidase expression construct (B-GAL) used was pcDNA3.1/His/ LacZ (Invitrogen).

### Transactivation assay

Reporter construct LEXA-fLUC was described previously [42]. Reporter gene HSP70-fLUC was obtained by subcloning promoter and RNA leader sequences of the human HSP70B gene into a plasmid containing a firefly luciferase gene. Constructs containing a constitutively expressed *Renilla *luciferase gene (pRL-TK, pRL-CMV) were obtained from Promega. Cultures in 96-well plates were transfected using a rapid transfection protocol for Lipofectamine 2000 (Gibco). Typically, each well received 0.75–1.0 μl of Lipofectamine 2000 in 25 μl Opti-MEM and a DNA master mixture (88.25 ng) in 25 μl Opti-MEM consisting of 80 ng of LEXA-fLUC or HSP70-fLUC, 0.25 ng of pRL-Tk or 0.1 ng of pRL-CMV, 0.1–0.5 ng of LEXA-HSF1 or HSF1 (or a mutant) and 7.5–8.05 ng of B-GAL. 80,000 cells in 100 μl DMEM were added subsequently. Typically, transfections were carried out in triplicate. Transfected cells were incubated for 16–20 hours, heat-treated at 44°C for 30 min (unless indicated otherwise) or not heat-treated and harvested 6–7 hours later. The length of the period between heat treatment and cell harvest was optimized for expression of firefly luciferase and recovery of *Renilla *luciferase activity. Luciferase activity was measured using the Dual Luciferase Kit (Promega) and a Stratec plate luminometer. Typically, luciferase activity assays were performed in triplicate.

### Electrophoretic mobility shift assay

Cells were transfected in 100 mm-dishes with Lipofectamine PLUS, 25 ng of HSF1 or mutant HSF1 expression construct and 2.975 μg B-GAL according to the manufacturer's instructions, incubated for 24 hours and then either heat-treated or left untreated. PBS-washed cells were resuspended in buffer C (20 mM Hepes, pH7.9, 0.42 M NaCl, 1.5 mM MgCl_2_, 0.2 mM EDTA, 0.5 mM dithiothreitol, "Complete" Protease Inhibitor Cocktail (Roche), 25% glycerol). Extracts were prepared by three cycles of quick-freezing and thawing, and clarification by centrifugation. The same protocol was also employed for preparing extracts used for native gel electrophoresis. Extracts were either used immediately or were stored at -70°C. Electrophoretic mobility shift assays were carried out essentially as described before [7]. Signals were detected and quantified using a Molecular Dynamics PhosphorImager.

### Subcellular fractionation

Cells were transfected as described under the previous section. Fractionation was performed using NE-PER Nuclear and Cytoplasmic Extraction Reagents from Pierce according to the manufacturer's protocol. Correct operation of the protocol was verified by following endogenous HSF1 whose localization had been determined previously.

### Analysis of HSF1 phosphopeptides and identification of phosphorylated residues

9.0 × 10^6 ^Hela-CAT cells in 150-mm dishes were transfected with 32 μg of FLAG-HSF1 expression construct. For experiments involving analysis of radiolabeled phosphopeptides, 20 hours after transfection each culture was washed once with 25 ml phosphate-free DMEM containing 5% FCS and was incubated for 1 hour in the same medium. Medium was replaced by fresh medium further containing 2 mCi of ^32^P-orthophosphate (NEX011, NEN), and cells were incubated for 3 hours at 37°C. After heat treatment for 45 min at 44°C, cells were washed with ice-cold PBS and lysed by incubation for 15 min at room temperature in 3 ml Mper buffer (Pierce) supplemented with 0.5 mM NaV_3_, 5 mM NaF, 150 mM NaCl, 1 μM ocadaic acid and "Complete" Protease Inhibitor Cocktail (Roche). After removal of debris, extract was incubated overnight at 4°C with 80 μl of FLAG M2 resin. Resin was washed extensively with Mper buffer containing 150 mM NaCl and Mper buffer alone, and FLAG-HSF1 was eluted with 120 μl of 6X SDS-PAGE sample buffer. Subsequent to electrophoresis on a Tris-Tricine high-resolution SDS-PAGE gel [43], FLAG-HSF1 was Coomassie-stained, and gel pieces containing the most highly phosphorylated species were processed by the Keck Foundation Biotechnology Resource Laboratory at Yale (Kenneth Williams) for proteolytic digestion, mass spectrometric analysis, and radiochemical and normal peptide sequencing. In other experiments, unlabeled, purified FLAG-HSF1 was prepared using a similar protocol. Sequence analysis of these preparations was performed at the Harvard Microchemistry Facility using microcapillary reverse-phase HPLC nano-electrospray tandem mass spectrometry on a Finnigan LCQ DECA quadrupole ion trap mass spectrometer.

### Other methods

For most immunoprecipitation and HSF1 expression experiments, cells were lysed in MPer-buffer supplemented with 150 mM NaCl and "Complete" Protease Inhibitor Cocktail from Roche. Protein concentrations in extracts were measured using a Bradford assay (Protein Assay reagent from Bio-Rad Laboratories). Results were used to adjust protein concentrations in extracts to be compared.

## Authors' contributions

TG carried out the bulk of the experimental work presented herein. He also prepared all tables and designed all figures. FB prepared a first set of LEXA-HSF1 substitution mutants. He also designed all oligonucleotide primers used in the study as well as analyzed all sequence information. WSL carried out or supervised all work concerning the identification of phosphorylated residues of HSF1 using LC/MS/MS, interpreted all data obtained and consulted on experimental design. RV conceived of the study, participated in its design and coordination, and drafted the manuscript. All authors read and approved the manuscript.

## References

[B1] Hunter T, Karin M (1992). The regulation of transcription by phosphorylation. Cell.

[B2] Hunter T (2000). Signaling – 2000 and beyond. Cell.

[B3] Sorger PK, Pelham HRB (1988). Yeast heat shock factor is an essential DNA-binding protein that exhibits temperature-dependent phosphorylation. Cell.

[B4] Sorger PK, Lewis MJ, Pelham HRB (1987). Heat shock factor is regulated differently in yeast and HeLa cells. Nature.

[B5] Sarge KD, Murphy SP, Morimoto RI (1993). Activation of heat shock gene transcription by heat shock factor 1 involves oligomerization, acquisition of DNA-binding activity, and nuclear localization and can occur in the absence of stress. Mol Cell Biol.

[B6] Green M, Schuetz TJ, Sullivan EK, Kingston RE (1995). A heat shock-responsive domain of human HSF1 that regulates transcription activation domain function. Mol Cell Biol.

[B7] Zuo J, Rungger D, Voellmy R (1995). Multiple layers of regulation of human heat shock transcription factor 1. Mol Cell Biol.

[B8] Cotto JJ, Kline M, Morimoto RI (1996). Activation of heat shock factor 1 DNA binding precedes stress-induced serine phosphorylation. Evidence for a multistep pathway of regulation. J Biol Chem.

[B9] Kline MP, Morimoto RI (1997). Repression of the heat shock factor 1 transcriptional activation domain is modulated by constitutive phosphorylation. Mol Cell Biol.

[B10] Bruce JL, Price BD, Coleman CN, Calderwood SK (1993). Oxidative injury rapidly activates the heat shock transcription factor but fails to increase levels of heat shock proteins. Cancer Res.

[B11] Xia W, Voellmy R (1997). Hyperphosphorylation of heat shock transcription factor 1 is correlated with transcriptional competence and slow dissociation of active factor trimers. J Biol Chem.

[B12] Chang NT, Huang LE, Liu AYC (1993). Okadaic acid markedly potentiates the heat-induced hsp 70 promoter activity. J Biol Chem.

[B13] Erdos G, Lee YJ (1994). Effect of staurosporine on the transcription of HSP70 heat shock gene in HT-29 cells. Biochem Biophys Res Commun.

[B14] Yamamoto N, Smith NW, Maki A, Berezeski IK, Trump BF (1994). Role of cytosolic Ca2+ and protein kinases in the induction of the hsp70 gene. Kidney Int.

[B15] Ohnishi K, Wang X, Takahashi A, Matsumoto H, Ohnishi T (1999). The protein kinase inhibitor, H-7, suppresses heat induced activation of heat shock transcription factor 1. Mol Cell Biochem.

[B16] Baek SH, Lee UY, Park EM, Han MY, Lee YS, Park YM (2001). Role of protein kinase Cdelta in transmitting hypoxia signal to HSF and HIF-1. J Cell Physiol.

[B17] Park J, Li AY (2001). JNK phosphorylates the HSF1 transcriptional activation domain: role of JNK in the regulation of the heat shock response. J Cell Physiol.

[B18] Fritsch M, Wu C (1999). Phosphorylation of Drosophila heat shock transcription factor. Cell Stress Chaperones.

[B19] Holmberg CI, Hietakangas V, Mikhailov A, Rantanen JO, Kallio M, Meinander A, Hellman J, Morrice N, MacKintosh C, Morimoto RI, Eriksson JE, Sistonen L (2001). Phosphorylation of serine 230 promotes inducible transcriptional activity of heat shock factor 1. EMBO J.

[B20] Soncin F, Zhang X, Chu B, Wang X, Asea A, Stevenson MA, Sacks DB, Calderwood SK (2003). Transcriptional activity and DNA binding of heat shock factor-1 involve phosphorylation on threonine 142 by CK2. Biochem Biophys Res Commun.

[B21] Hoj A, Jacobsen BK (1994). A short element required for turning off heat shock transcription factor: evidence that phosphorylation enhances deactivation. EMBO J.

[B22] Mivechi NF, Giaccia AJ (1995). Mitogen-activated protein kinase acts as a negative regulator of the heat shock response in NIH3T3 cells. Cancer Res.

[B23] Chu B, Zhong R, Soncin F, Stevenson MA, Calderwood SK (1998). Transcriptional activity of heat shock factor 1 at 37 degrees C is repressed through phosphorylation on two distinct serine residues by glycogen synthase kinase 3 and protein kinases Calpha and Czeta. J Biol Chem.

[B24] Chu B, Soncin F, Price BD, Stevenson MA, Calderwood SK (1996). Sequential phosphorylation by mitogen-activated protein kinase and glycogen synthase kinase 3 represses transcriptional activation by heat shock factor-1. J Biol Chem.

[B25] Knauf U, Newton EM, Kyriakis J, Kingston RE (1996). Repression of human heat shock factor 1 activity at control temperature by phosphorylation. Genes Dev.

[B26] Newton EM, Knauf U, Green M, Kingston RE (1996). The regulatory domain of human heat shock factor 1 is sufficient to sense heat stress. Mol Cell Biol.

[B27] He B, Meng YH, Mivechi NF (1998). Glycogen synthase kinase 3beta and extracellular signal-regulated kinase inactivate heat shock transcription factor 1 by facilitating the disappearance of transcriptionally active granules after heat shock. Mol Cell Biol.

[B28] Xia W, Guo Y, Vilaboa N, Zuo J, Voellmy R (1998). Transcriptional activation of heat shock factor HSF1 probed by phosphopeptide analysis of factor ^32^P-labeled in vivo. J Biol Chem.

[B29] Dai R, Frejtag W, He B, Zhang Y, Mivechi NF (2000). c-Jun NH2-terminal kinase targeting and phosphorylation of heat shock factor-1 suppress its transcriptional activity. J Biol Chem.

[B30] Xavier IJ, Mercier PA, McLoughlin CM, Ali A, Woodgett NJR, Ovsenek N (2000). Glycogen synthase kinase 3beta negatively regulates both DNA-binding and transcriptional activities of heat shock factor 1. J Biol Chem.

[B31] McMillan DR, Xiao X, Shao L, Graves K, Benjamin IJ (1998). Targeted disruption of heat shock transcription factor 1 abolishes thermotolerance and protection against heat-inducible apoptosis. J Biol Chem.

[B32] Wang X, Grammatikakis N, Siganou A, Stevenson MA, Calderwood SK (2004). Interactions between extracellular signal regulated protein kinase 1 (ERK), 14-3-3 epsilon and heat shock factor 1 during stress. J Biol Chem.

[B33] Hietakangas V, Ahlskog JK, Jakobsson AM, Hellesuo M, Sahlberg NM, Holmberg CI, Mikhailov A, Palvimo JJ, Pirkkala L, Sistonen L (2003). Phosphorylation of serine 303 is a prerequisite for the stress-inducible SUMO modification of heat shock factor 1. Mol Cell Biol.

[B34] Huang L, Mivechi NF, Moskophidis D (2001). Insights into regulation and function of the major stress-induced hsp70 molecular chaperone in vivo: analysis of mice with targeted gene disruption of the hsp70.1 or hsp70.3 gene. Mol Cell Biol.

[B35] Xie Y, Zhong R, Chen C, Calderwood SK (2003). Heat shock factor 1 contains two functional domains that mediate transcriptional repression of the c-fos and c-fms genes. J Biol Chem.

[B36] Jones TJ, Li D, Wolf IM, Wadekar SA, Periyasamy S, Sanchez ER (2004). Enhancement of glucocorticoid receptor-mediated gene expression by constitutively active heat shock factor 1. Mol Endocrinol.

[B37] Stephanou A, Latchman DS (1999). Transcriptional regulation of the heat shock protein genes by STAT family transcription factors. Gene Expr.

[B38] Xie Y, Chen C, Stevenson MA, Auron PE, Calderwood SK (2002). Heat shock factor 1 represses transcription of the IL-1beta gene through physical interaction with the nuclear factor of interleukin 6. J Biol Chem.

[B39] Blom N, Gammeltoft S, Brunak S (1999). Sequence- and structure-based prediction of eukaryotic protein phosphorylation sites. J Mol Biol.

[B40] Hsu AL, Murphy CT, Kenyon C (2004). Regulation of aging and age-related disease by DAF-16 and heat-shock factor. Science.

[B41] Morley JF, Morimoto RI (2004). Regulation of longevity in Coenorhabditis elegans by heat shock factor and molecular chaperones. Mol Biol Cell.

[B42] Guo Y, Guettouche T, Fenna M, Boellmann F, Pratt WB, Toft DO, Smith DF, Voellmy R (2001). Evidence for a mechanism of repression of heat shock factor 1 transcriptional activity by a multichaperone complex. J Biol Chem.

[B43] Schagger H, von Jagow G (1987). Tricine-sodium dodecyl sulfate-polyacrylamide gel electrophoresis for the separation of proteins in the range from 1 to 100 kDa. Anal Biochem.

